# Comparative anatomy of the *Sapajus* sp. (bearded capuchin) hand with comments on tool use in a parallel evolution with the hominid pathway

**DOI:** 10.3389/fphys.2024.1292035

**Published:** 2024-02-09

**Authors:** Rafael Bretas, Emmanuel Freitas-Ferreira, Rafael Souto Maior, Carlos Tomaz, Maria Tereza Gonçalves-Mendes, Tales Alexandre Aversi-Ferreira

**Affiliations:** ^1^ Innovation Design Office, Riken, Kobe, Japan; ^2^ Institute of Exact Sciences, Federal University of Alfenas, Alfenas, Brazil; ^3^ Laboratory of Physiological Sciences, Department of Structural Biology, Institute of Biological Sciences, University of Brasilia, Brasilia, Brazil; ^4^ Postgraduate Program in Environment, University CEUMA, São Luís, Brazil; ^5^ Laboratory of Biomathematics, Institute of Biomedical Sciences, Federal University of Alfenas, Alfenas, Brazil

**Keywords:** *Sapajus*, primate hand, morphology of recent groups, biomathematics, mathematical models, evolution, primates

## Abstract

**Introduction:** Bearded capuchins display a wide variety of manipulatory skills and make routine use of tools in both captivity and the wild. The efficient handling of objects in this genus has led several investigators to assume near-human thumb movements, despite a lack of anatomical studies.

**Methods:** Here, we performed an anatomical analysis of muscles and bones in the capuchin hand. *Sapajus* morphological traits were quantitatively compared with those of humans, chimpanzees, gorillas, and baboons.

**Results:** The comparative analysis indicated that the *Sapajus* hand is more similar to that of baboons and least similar to that of humans according to the muscles, bones, and three-dimensional data. Furthermore, these findings suggest that bearded capuchins lack true thumb opponency. Regarding manipulatory skills, they display rather primitive hand traits, with limited resources for precision grasping using the opponens pollicis.

**Discussion:** These findings suggest that bearded capuchins’ complex use of tools depends more heavily on their high cognitive abilities than on a versatile hand apparatus. These findings offer crucial insights into the evolution of primate cognition.

## 1 Introduction

Despite being a New World primate, bearded capuchins (*Sapajus* sp.) exhibit several evolutionary convergences with great apes and humans, including a long lifespan (Hakeem et al., 1996; Judge and Carey, 2000), a well-developed neocortex (Rilling and Insel, 1999), high degrees of gregariousness and social tolerance (Fragazy et al., 2004), and tool use in both captivity and the wild (Canale et al., 2009; Mannu and Ottoni, 2009; Spagnoletti et al., 2009; Souto et al., 2011). Nevertheless, in other anatomical characteristics, such as anatomical patterns of vessels, nerves, and muscles of the thoracic and pelvic limbs, capuchins are more similar to baboons (Aversi-Ferreira et al., 2013; Aversi-Ferreira et al., 2014c; Aversi-Ferreira et al., 2020; Aversi-Ferreira et al., 2021a; Abreu et al., 2021) and New World primates (Aversi-Ferreira et al., 2010).

A detailed study demonstrated that despite performing precision grip and high manual abilities, bearded capuchins present a primitive anatomical structure in their hands from the forearm muscles, as opposed to those found in great apes and humans (i.e., bearded capuchins do not realize true thumb opponency); therefore, they putatively use their large brain to generate strategies to contour over their primitive apparatus (Aversi-Ferreira et al., 2014a; Abreu et al., 2021). Indeed, the use of complex tools requires high motor and cognitive skills as well as an appropriate hand apparatus, in support of which *Sapajus* presents high indices of encephalization (Fragazy et al., 2004; Pereira-de-Paula et al., 2010; Borges et al., 2015; Aversi-Ferreira et al., 2021a; Abreu et al., 2021) and abundant corticospinal motor connections (Stephan and Andy, 1969; Rilling and Insel, 1999; Abreu et al., 2021) to support the planning and coordination involved in tool use.

Behavioral accounts of capuchins’ hand abilities have been asserted despite the lack of complete and/or detailed anatomical analyses of their hand and forearm structures. One exception to this was a study concerning bearded capuchins’ hand joints, bones, and thumb muscles (Aversi-Ferreira et al., 2014a). It demonstrated various strategies for the precision grip and lateral pinch of *Sapajus* and fine movements found in certain human performance, such as holding a pen or pencil to write. Moreover, studies on bearded capuchins’ forearms (Aversi-Ferreira et al., 2010; Aversi-Ferreira et al., 2011) have demonstrated the presence of a separated tendon of the extensor pollicis longus and extensor indicis in *Sapajus*, which until then had only been observed in modern humans (Aversi-Ferreira et al., 2010).

The aforementioned particularities of bearded capuchins concern similarities with great apes in cognitive and behavioral aspects as well as in certain muscles; on the other hand, morphological similarities exist with baboons and New World primates. Therefore, we performed dissections on bearded capuchin hands, describing the muscles involved in hand movements, as well as their origins, insertions, innervation, supply, and number. These data were compared with the anatomical descriptions of modern humans (Swindler and Wood, 1973; Gibbs, 1999; Standring, 2008), chimpanzees (Swindler and Wood, 1973; Gibbs, 1999), gorillas (Swindler and Wood, 1973; Gibbs, 1999), and baboons (Swindler and Wood, 1973), and the results were associated with behavioral and tool use aspects. All descriptions were quantified and compared using the statistical method for a comparative anatomy (Aversi-Ferreira, 2009; Aversi-Ferreira et al., 2011; Aversi-Ferreira et al., 2014a; Aversi-Ferreira et al., 2014b; Aversi-Ferreira et al., 2015; Aversi-Ferreira et al., 2016).

## 2 Materials and methods

### 2.1 Samples

Eight adult *Sapajus* specimens were used (six males and two females), weighing 1–3 kg. No animals were killed for the purpose of this study; four of them suffered accidental deaths in their natural habitats and were acquired from the anatomical collection of the Laboratory of Neurosciences and Behavior (LNB) at the Department of Physiological Sciences, Institute of Biology, University of Brasília. The remaining four specimens belonged to the Brazilian Institute of Environment and Renewable Natural Resources (IBAMA) archive and were donated to the University of Uberlândia in the 1970s before being donated to the LNB. This study was approved by the Institutional Ethical Committee (CoEP-UFG 81/2008, with authorization from IBAMA number 15275).

### 2.2 Preparation of animals for dissection

All procedures involving the animals were performed in accordance with the guidelines of the Brazilian Society of Animal Experimentation (COBEA). After trichotomy using a razor blade, the animals were incubated in water at room temperature for 10–12 h; then, they received perfusion through the femoral vein with 10% formaldehyde and 5% glycerin for fixation. The animals were conserved in 10% formaldehyde in covered opaque cubes to avoid light penetration and the evaporation of formaldehyde.

### 2.3 Dissection and documentation

Hand dissection was performed on six subjects to expose their muscles, while the other two were prepared to study their hand joints and bones. All materials were registered using a digital camera, schematic drawings, and anatomical descriptions. The nomenclature of the hand muscles follows, wherever possible, the guidelines used by Gibbs (1999). The data collected were analyzed and compared with the patterns described for human, chimpanzee, gorilla, and baboon species.

### 2.4 Statistical analysis

The statistical analysis was based on the previous methods described by Aversi-Ferreira and colleagues (Aversi-Ferreira, 2009; Aversi-Ferreira et al., 2011; Aversi-Ferreira et al., 2014a; Aversi-Ferreira et al., 2014b; Aversi-Ferreira et al., 2015; Aversi-Ferreira et al., 2016). The methods used to calculate the similarities in the hand muscles among different species are summarized in Table 1. In this work, the bearded capuchin was primarily chosen as the reference (control species) for comparison with other primates, although they were also analyzed among themselves (Tables 2, 3, 5).

**TABLE 1:** General methods for calculating the similarities of muscles between *Sapajus* and other species (i.e., baboon, apes, and modern humans).

Species		Bearded capuchin (control species; *i* = 1)					Baboon (*i* = 2)				…	Modern human (*i* = 5)			
Investigated structure		Contrahentes		Lumbricals	…	Dorsal interossei	Contrahentes	Lumbricals	…	Dorsal interossei	…	Contrahentes	Lumbricals	…	Dorsal interossei
P_*ijk*_		Specific weights given to the variations													
Innervation (k = 1)		w_1_ (=3)	P_111_	P_121_	…	P_1m1_	P_211_	P_221_	…	P_2m1_	…	P_s11_	P_s21_	…	P_sm1_
Origin (k = 2)		w_2_ (=2)	P_112_	P_122_	…	P_1m2_	P_212_	P_222_	…	P_2m2_	…	P_s12_	P_s22_	…	P_sm2_
Insertion (k = 3)		w_3_ (=2)	P_113_	P_123_	…	P_1m3_	P_213_	P_223_	…	P_2m3_	…	P_s13_	P_s23_	…	P_sm3_
Vascularization (k = 4)		w_4_ (=1)	P_114_	P_124_	…	P_1m4_	P_214_	P_224_	…	P_2m4_	…	P_s14_	P_s24_	…	P_sm4_
Number of muscles for each arm (k = 5)		w_5_ (=1)													
Weighted averages for a single muscle (PAF = *P*_*w(ij)*_)			*P*_*w(11)*_	*P*_*w(12)*_	…	*P*_*w(1m)*_	*P*_*w(21)*_	*P*_*w(22)*_	…	*P*_*w(2m)*_	…	*P*_*w(s1)*_	*P*_*w(s2)*_	…	*P*_*w(sm)*_
Weighted averages for multiple muscles (mean of *P*_*w(ij)*_) (*P*_*w(i)*_)			*P*_*w(1)*_				*P*_*w(2)*_				…	*P*_*w(s)*_			
GCAI = |*P*_*w(i)*_ - *P*_*w(i’)*_|							|*P*_*w(1)*_−*P*_*w(2)*_|				…	|*P*_*w(1)*_−*P*_*w(s)*_|			

**TABLE 2:** Partial modal data of anatomical structures with some information about the structures that generate the data for CAI calculations.

Species structure	*Sapajus*		Human	Chimpanzee		Gorilla	*Papio*
Central portion							
1. *Contrahentes muscles*	Innervation	Ulnar nerve (16/16)	0	Identical to the model	0		Identical to the model
	Origin	All muscles originated from the proximal portion of the flexor retinaculum (16/16)	0	Identical to the model	0		Identical to the model
	Insertion	Base of digits II–V, respectively (16/16)	0	Identical to the model	0		Identical to the model
	Arterial supply	Palmar arch (16/16)	0	Both palmar arches	0		Identical to the model
	Number of muscles in the species	4	0	2	0		3
2. *Lumbricals*	Innervation	I and II: median nerve; III and IV: ulnar nerve (12/16)	Identical to the model	Identical to the model	Identical to the model		Identical to the model
	Origin	Flexor digitorum profundus tendons under the flexor retinaculum (16/16)	Identical to the model	Identical to the model	I: flexor pollicis longus tendon		Identical to the model
	Insertion	Lateral side of the digital extensor from II to V fingers (16/16)	Identical to the model	Identical to the model	Identical to the model		Identical to the model
	Arterial supply	Palmar arch (16/16)	Both palmar arches	Both palmar arches	Both palmar arches		Identical to the model
	Number of muscles in the species	4	4	4	4		4
3. *Palmar interossei*	Innervation	Ulnar nerve (16/16)	Identical to the model	Identical to the model	Identical to the model		Identical to the model
	Origin	(16/16)	Identical to the model	Identical to the model	Identical to the model		Identical to the model
		I: lateral margin of metacarpal II					
		II: medial margin of metacarpal II and flexor retinaculum					
		III: medial margin of metacarpal IV					
		IV: medial margin of metacarpal V					
	Insertion	(16/16)	Identical to the model	Identical to the model	Identical to the model		Identical to the model
		I: proximal phalanx of digit II, medial portion					
		II: base of the proximal phalanx of digit III and metacarpophalangeal joint					
		III: base of the proximal phalanx of digit IV and metacarpophalangeal joint					
		IV: base of the proximal phalanx of digit V					
	Arterial supply	Ulnar artery branches (16/16)	Identical to the model	Identical to the model	Identical to the model		Identical to the model
	Number of muscles in the species	4	4	6–7	5		7
4. *Dorsal interossei*	Innervation	Ulnar nerve (16/16)	Identical to the model	Identical to the model	Identical to the model		Identical to the model
	Origin	(16/16)	Identical to the model	Identical to the model	I: additional origin from the pyramidal bone		Identical to the model
		I: metacarpal I and II					
		II: metacarpal II and III					
		III: metacarpal III and IV					
		IV: metacarpal IV and V					
	Insertion	(16/16)	Identical to the model	IV: inserts onto the lateral side of metacarpal V	Identical to the model		Identical to the model
		I: digit II at the lateral side of the proximal phalanx					
		II: digit II at the medial side of the proximal phalanx					
		III: digit III at the medial side of the proximal phalanx					
		IV: digit IV at the medial side of the proximal phalanx					
	Arterial supply	Palmar arch (16/16)	Both palmar arches	Both palmar arches	Both palmar arches		Identical to the model
	Number of muscles in the species	4	4	4	4		4
Hypothenar muscles							
5. *Palmaris brevis*	Innervation	Ulnar nerve (16/16)	Identical to the model	Identical to the model	Identical to the model		Identical to the model
	Origin	Palmar aponeurosis (16/16)	Palmar aponeurosis and annular ligament	Identical to the model	Palmar aponeurosis, annular ligament, and pisiform		Identical to the model
	Insertion	Medial part of the hand skin (16/16)	Identical to the model	Identical to the model	Identical to the model		Identical to the model
	Arterial supply	Ulnar artery (16/16)	Identical to the model	Identical to the model	Identical to the model		Identical to the model
	Number of muscles in the species	1	1	1	1		1
6. *Abductor digiti minimi*	Innervation	Ulnar nerve (16/16)	Identical to the model	Identical to the model	Identical to the model		Identical to the model
	Origin	Pisiform and hamulus of hamate and medial portion of the flexor retinaculum (16/16)	Pisiform, carpal ulnar ligament, pisohamate ligament, and tendon of the flexor carpi ulnaris	Pisiform and carpal ulnar ligament	Does not present an origin from the hamulus of the hamate		Pisiform and carpal ulnar ligament
	Insertion	Digit V at the medial base of the proximal phalanx and metacarpophalangeal capsule (16/16)	Identical to the model	Identical to the model	Identical to the model		Identical to the model
	Arterial supply	Ulnar artery (16/16)	Identical to the model	Identical to the model	Identical to the model		Identical to the model
	Number of muscles in the species	1	1	1	1		1
7. *Flexor digiti minimi brevis*	Innervation	Ulnar nerve (16/16)	Identical to the model	Identical to the model	Identical to the model		Identical to the model
	Origin	Flexor retinaculum (16/16)	Originates at the hamulus of the hamate and flexor retinaculum	Originates at the hamulus of the hamate and flexor retinaculum	Originates at the hamulus of the hamate and flexor retinaculum. Additional insertion onto the capsule of the metacarpophalangeal joint		Originates at the hamulus of the hamate and flexor retinaculum
	Insertion	Digit V at the base of the proximal phalange (16/16)	Identical to the model	Identical to the model	Identical to the model		Identical to the model
	Arterial supply	Ulnar artery branches (16/16)	Identical to the model	Identical to the model	Identical to the model		Identical to the model
	Number of muscles in the species	1	1	1	1		1
8. *Opponens digiti minimi*	Innervation	Ulnar nerve (16/16)	Identical to the model	Identical to the model	Identical to the model		Identical to the model
	Origin	Hamulus of the hamate, pisiform bone, and flexor retinaculum (16/16)	Hamulus of the hamate and flexor retinaculum	Hamulus of the hamate and flexor retinaculum	Hamulus of the hamate and flexor retinaculum		Hamulus of the hamate and flexor retinaculum
	Insertion	Medial margin at the distal end of metacarpal V and the metacarpophalangeal capsule (16/16)	Identical to the model	Identical to the model	Identical to the model		Identical to the model
	Arterial supply	Ulnar artery (16/16)	Identical to the model	Identical to the model	Identical to the model		Identical to the model
	Number of muscles in the species	1	1	1	1		1
Thenar muscles							
9. *Abductor pollicis brevis*	Innervation	Ulnar nerve (16/16)	Identical to the model and branches from the median nerve	Identical to the model	Identical to the model		Identical to the model
	Origin	Palmar aponeurosis, flexor retinaculum, and scaphoid bone (16/16)	Origin at the trapezium and scaphoid^†^ and flexor retinaculum	Trapezium bone and flexor retinaculum	Trapezium bone and flexor retinaculum		Trapezium bone and flexor retinaculum
	Insertion	Digit I at the lateral base of the proximal phalanx (16/16)	Identical to the model	Identical to the model	Identical to the model		Identical to the model
	Arterial supply	Radial artery (16/16)	Identical to the model	Identical to the model	Identical to the model		Identical to the model
	Number of muscles in the species	1	1	1	1		1
10. *Flexor pollicis brevis*	Innervation	Median nerve (16/16)	Radial head: median nerve; ulnar head: ulnar nerve	Identical to the model	Identical to the model		Identical to the model
	Origin	Medial portion at the distal end of the palmar aponeurosis and distal portion of the flexor retinaculum (16/16)	Two heads that originate from the flexor retinaculum and tuberculum of trapezium, trapezoid, and capitate	Similar to humans. Ulnar head is present in 3/12	Similar to humans. Ulnar head is present in 1/16		Origin at the trapezoid and metacarpal II
	Insertion	Digit I at the antero-medial side of the proximal phalanx and articular capsule (16/16)	Identical to the model	Identical to the model	Identical to the model		Identical to the model
	Arterial supply	Radial artery (16/16)	Identical to the model	Identical to the model	Identical to the model		Identical to the model
	Number of muscles in the species	1	1	1	1		1
11. *Opponens pollicis*	Innervation	Median nerve (16/16)	Identical to the model	Identical to the model	Identical to the model		Identical to the model
	Origin	Trapezium and trapezium–metacarpal I articular capsule (16/16)	Identical to the model	Identical to the model	Identical to the model		Identical to the model
	Insertion	Anterior side of metacarpal I and the base of the metacarpophalangeal joint (16/16)	Lateral side of metacarpal 1	Similar to humans	Similar to humans		Similar to humans
	Arterial supply	Radial artery (16/16)	Identical to the model	Identical to the model	Identical to the model		Identical to the model
	Number of muscles in the species	1	1	1	1		1
12. *Adductor pollicis*	Innervation	Ulnar nerve (16/16)	Identical to the model	Identical to the model	Identical to the model		Identical to the model
	Origin	Oblique head: metacarpal II diaphysis, capitate, and scaphoid. Transverse head: distal end of metacarpal III (16/16)	Origin at capitate and metacarpal II and III	Similar to humans	Origin at the base of metacarpals I, II, and/or III; tendon of contrahentes; and metacarpophalangeal joints II and III		Origin at the base of metacarpals II and III; tendon of contrahentes; and metacarpophalangeal joints II and III
	Insertion	Digit I at the medial portion of the base of the proximal phalanx (16/16)	Identical to the model	Identical to the model	Identical to the model		Identical to the model
	Arterial supply	Radial artery (16/16)	Identical to the model	Identical to the model	Identical to the model		Identical to the model
	Number of muscles in the species	1	1	1	1		1

**TABLE 3:** Muscles of the central portion of the capuchin hand with origins and insertions compared with modern humans, apes, and baboons.

Central portion	Origin	Insertion	Human	Chimpanzee	Gorilla	Baboon
1. *Contrahentes*	Proximal portion of the flexor retinaculum	Base of digits II–V, respectively	Absent. CAI = 1	Reduced and associated with the aponeurosis. CAI = 0.111	CAI = 1	CAI = 0.0278
2. *Lumbricals*	Flexor digitorum profundus tendons, under the flexor retinaculum	Lateral side of the digital extensor from II to V fingers	Identical origin and insertions. CAI = 0.056	Identical origin and insertions. CAI = 0.056	First originates also from the flexor pollicis longus. Identical insertions. CAI = 0.139	Identical origin and insertions. CAI = 0
3. *Palmar interossei*	I: lateral margin of metacarpal II	I: proximal phalanx of digit II and the medial portion	Presents three palmar interosseous muscles with divergent origins. Identical insertions. CAI = 0.083	Presents six or seven muscles. Identical origins and insertions. CAI = 0.125	Presents five muscles. Identical origins and insertions. CAI = 0.083	Presents seven, origin in two heads of the adjacent metacarpal. Identical insertions. CAI = 0.31
	II: medial margin of metacarpal II and the flexor retinaculum	II: base of the proximal phalanx of digit III and the metacarpophalangeal joint				
	III: medial margin of metacarpal IV	III: base of the proximal phalanx of digit IV and the metacarpophalangeal joint				
	IV: medial margin of metacarpal V	IV: base of the proximal phalanx of digit V				
4. *Dorsal interossei*	I: metacarpal I and II	I: digit II at the lateral side of the proximal phalanx	Identical origins and insertions. CAI = 0.056	Fourth dorsal interosseous inserts onto the lateral side of metacarpal V. Identical insertions. CAI = 0.111	First dorsal interosseous muscle also originates from the pyramidal bone. Identical insertions. CAI = 0.111	Identical origins and insertions. CAI = 0
	II: metacarpal II and III	II: digit II at the medial side of the proximal phalanx				
	III: metacarpal III and IV	III: digit III at the medial side of the proximal phalanx				
	IV: metacarpal IV and V	IV: digit IV at the medial side of the proximal phalanx				
Central group			GCAI = 0.299	GCAI = 0.101	GCAI = 0.333	GCAI = 0.085

The values in Table 1 are calculated based on the following formula:

$$P_{ijk}=\frac{r_{v\left(ijk\right)}}{N},$$

where ***P*** is the relative frequency, ***N*** is the total number of analyzed structures of the samples (Table 2), and ***r***_***v***_ is the number of normal structures.

To account for the influence of the weight on the relationship with the body size, Aversi-Ferreira et al. (2015) proposed the method of assigning weights based on the degree of evolutionary divergence. Accordingly, innervation was assigned a weight of 3, origin and insertion a weight of 2, vascularization a weight of 1, and the number of muscles per hand a weight of 1. This weighting scheme prioritizes features with less variation across the evolutionary scale, resulting in a CAI calculation that emphasizes phylogenetic relationships between the compared animals.

When the number of insertions, origins, and/or muscles differed from those of the bearded capuchin’s, the value of P_ijk_ was calculated using the difference of 1 and a multiple of a ¼ of the number assigned to the control species (i.e., bearded capuchin). This multiple was the number of the structure greater or less than that ascribed to the control species. For instance, if the frequency P_ijk_ of the control species was 1, then the origin of a muscle in bearded capuchins occurred from only one structure, and for other species, it occurred from three structures; the calculation of P_ijk_ would be as follows:

$$P_{ijk}=1-\left[\frac{1}{4}∙\left|k_{Sapajus}-k_{other}\right|\right]=1-\left[\frac{1}{4}∙\left|1-3\right|\right]=1-\left[\frac{1}{4}∙\left|-2\right|\right]=1-\frac{1}{2}=\frac{1}{2}$$

for $k_{Sapajus}-k_{other}\geq 1$ . For $k_{Sapajus}-k_{other}\;<1$ , for instance, if a muscle had two heads with a frequency of 1 for a head and ¼ for the other, the $P_{ijk}$ value would be calculated as follows:

$$P_{ijk}=\left[\left|k_{Sapajus}-k_{other}\right|\right]=\left[\left|1-\frac{3}{4}∙\frac{1}{4}\right|\right]=\frac{13}{16}.$$

This procedure was necessary because when it is not possible to obtain data directly from an animal, the consulted literature does not always supply information about the frequency of the muscles but rather provides the variations in the number, origin, or insertion:

$$PAF=P_{w\left(ij\right)}=\frac{\sum_{k=1}^{q}w_{k}∙P_{ijk}}{\sum_{k=1}^{q}w_{k}}.$$

Here, PAF is the relative frequency and $w_{k}$ is the weighted coefficient attached to a given parameter (innervation, origin, insertion, vascularization, or the number of muscles in each hand). Notably, $w_{k}$ considers whether single or multiple muscles exist. For example, while *Sapajus* possesses four contrahentes in each hand, chimpanzees have only two.

$${CAI}_{ii’}=\left|P_{w\left(ij\right)}-P_{w\left(i’j’\right)}\right|,$$

where i≠i’ . ${CAI}_{ii’}$ represents an absolute difference in the weighted averages ( $P_{w\left(ij\right)}$ ) of a single structure between the *control species* ( i ) and other noncontrol ( i’ ) species. To compare one structure ( j=1 ) with one parameter ( k=1 ) between the control ( i=1 ) and noncontrol ( i’=2 ) species, the formula is modified as follows:

$${CAI}_{12}=\left|P_{w\left(11\right)}-P_{w\left(21\right)}\right|,$$

where i=1 and i’=2 .

Notably, ${CAI}_{ii’}$ ranges from 0 to 1 , that is, $0\leq {CAI}_{ii’}\leq 1$ . This is because the maximum value of $P_{w\left(ij\right)}$ is 1 and the minimum is 0 .

$$P_{w\left(1\right)}=\frac{\sum_{j=1}^{m_{j}}P_{w\left(ij\right)}}{m_{j}}.$$

Here, $m_{j}$ is the number of studied structures; in this work, m=4 for each portion of the hand, i.e., four muscles in the central part, four in the thenar part, and four in the hypothenar parts.

$${GCAI}_{ii‘}=\left|P_{w\left(i\right)}-P_{w\left(i‘\right)}\right|$$

or

$${GCAI}_{ii‘}=\left|\frac{\sum_{j=1}^{m_{j}}P_{w\left(ij\right)}}{m_{j}}-\frac{\sum_{j=1}^{m_{j}}P_{w\left(i‘j\right)}}{m_{j}}\right|.$$

Here, GCAI represents a value that indicates the distance of the group of the hand muscles in a given species from those of bearded capuchins’ (control species). The anatomical position of the human hand was employed as a reference for the descriptions.

In general terms, to calculate CAI, the same pattern of innervation was considered for *Sapajus*, baboons, chimpanzees, and humans based on the study by Marin and colleagues (2009), while that for gorillas was based on Gibbs (1999). Vascularization was deemed identical between bearded capuchins and baboons and different between these and the remaining species considered, following Aversi-Ferreira (2009), Aversi-Ferreira and colleagues (2020), Gibbs (1999), and Swindler and Wood (1973). In this sense, baboons and bearded capuchins have only one palmar arch in the hand, whereas apes and modern humans have two; thus, the frequency of vascularization (P_ijk_) for bearded capuchins and baboons was 1, while it was ½ for the other species.

## 3 Results and discussion

### 3.1 Superficial description

**FIGURE 1:**
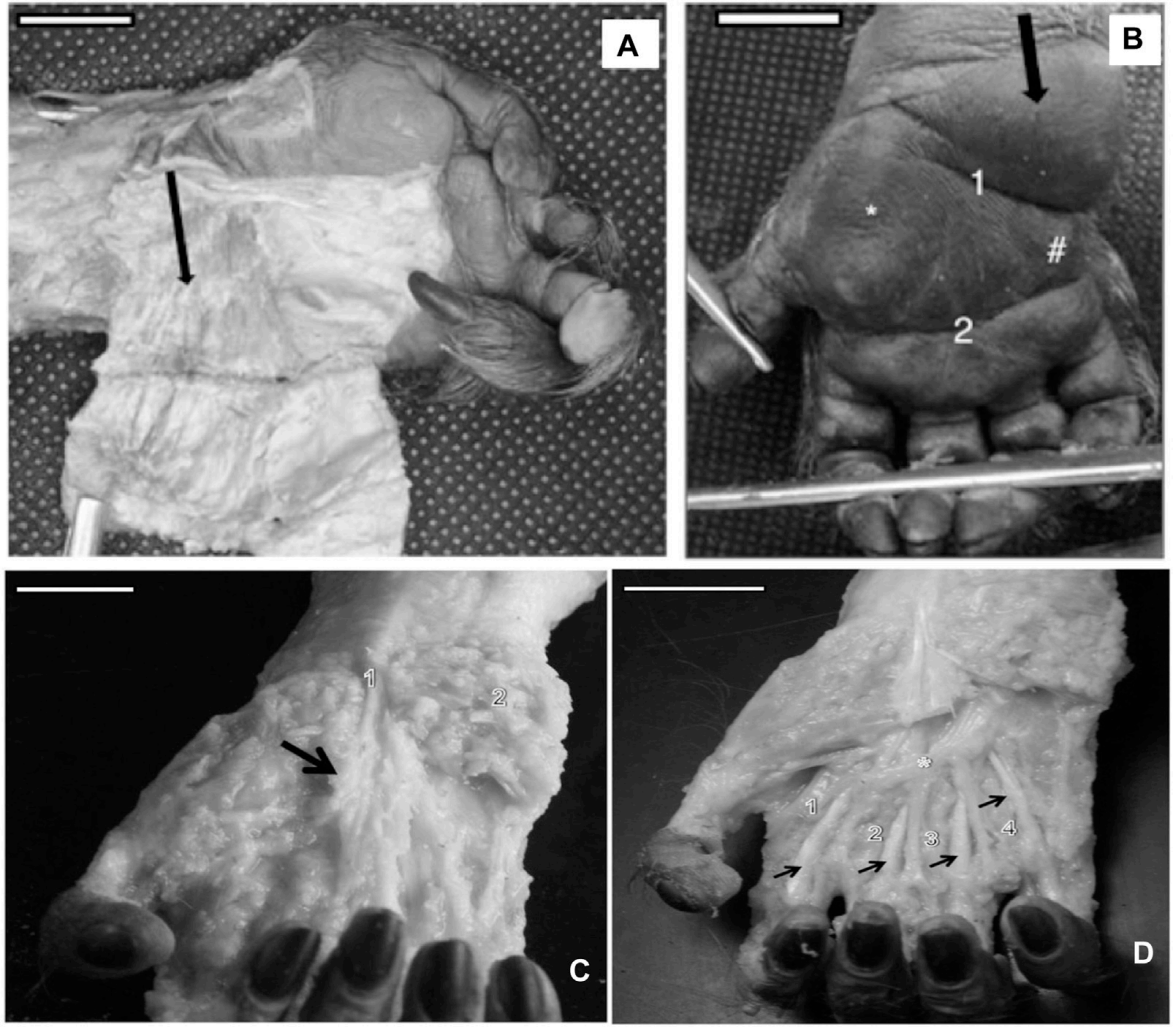
**(A)** Left hand of a bearded capuchin showing the dissected proximal hypothenar eminence (black arrow) and fibers of the muscle palmaris brevis (bar = 1.5 cm). **(B)** Black arrow indicates the nondissected proximal hypothenar eminence, * indicates the thenar eminence, # indicates the distal hypothenar eminence, 1 is the flexure proximal that separates the proximal from the distal eminence, and 2 indicates the simian flexure (bar = 1.3 cm). **(C)** Dissected right hand of a bearded capuchin exhibiting aponeurosis (arrow), a visible collagen bundle of the palmaris longus tendon (1), and the tissues of hypothenar eminence (2). Bar = 2.2 cm. **(D)** Dissected right hand of a bearded capuchin showing contrahentes (1, 2, 3, and 4) inserted in digits II–V and lying between the superficial and deep muscles (arrows). The asterisk (*) indicates the palmar arch unique to *Sapajus* (bar = 2.8 cm).

The bearded capuchin hand presents an expressive proximal hypothenar eminence with the majority placed proximally to the line between the styloid process of the radius and ulna, with lesser distal hypothenar and thenar eminences (Figures 1A, B). After removing the skin, we observed much dense unmodeled connective tissue forming the eminences and also the palmar aponeurosis with bundles of collagen, which are associated with the tendons of muscles that insert on the hand, such as the palmaris longus (Figure 1C). On the medial side, it was possible to distinguish muscular fibers of the muscle palmaris brevis (Figure 1A). The size of the proximal hypothenar eminence indicated a place used to support locomotion-caused friction and also for prehension, as observed in other primates (Swindler and Wood, 1973).

Furthermore, the thenar and distal hypothenar eminences were not as expressive as the proximal hypothenar eminences, presenting less connective tissues; however, it seemed to be associated with support for grabbing peanuts (Spinozzi et al., 2004).

Moreover, the flexure lines in the bearded capuchin hand displayed a different pattern, as cited by Swindler and Wood (1973), in relation to other primates; that is, the interdigital eminences observed in *Papio* were not present; the simian line did not terminate or take direction for the second interdigital space, as in modern humans, *Pan*, and *Papio*; and no deep longitudinal flexure lines were observed. Two transversal lines were evident in *Sapajus* hands, namely, a proximal line that separates the proximal from distal hypothenar eminences and terminates on the proximal part of the thenar eminence and a distal eminence—the simian line that crosses all palms.

Lastly, the primitive pattern of pentadactylism with a basic phalangeal formula (i.e., 3 >4 >2 >5 >1) observed in primates (Gibbs, 1999) was verified in the *Sapajus* hands.

### 3.2 Muscles

The intrinsic muscles of the hand are divided into the thenar, hypothenar, and central groups (Swindler and Wood, 1973). While the thenar and hypothenar muscles are associated with the thumb and digit V, respectively, the central group is associated with all fingers. Nevertheless, all muscle groups originate in the central proximal area of the hand.

#### 3.2.1 Central muscles of the hand

The *contrahentes* muscles act as a superficial layer in the palm, immediately below the palmar aponeurosis level (Table 3). All bearded capuchin specimens analyzed in this study presented four contrahentes in their hands. They originated in the proximal portion of the retinaculum of the flexor muscles and were inserted in the base of digits II, III, IV, and V. Their position lays between the superficial and deep flexor muscles of the fingers (Figure 1D). They were innervated through the ulnar nerve.

Humans do not present contracting muscles, while baboons have three and chimpanzees have only two that are reduced and aponeurotic, lacking the digit II contrahens muscle; otherwise, all have the same origin in the species (Swindler and Wood, 1973). Gorillas, on the other hand, do not display distinct contrahens muscles (Gibbs, 1999). According to Swindler and Wood (1973), the contrahentes are innervated through the ulnar nerve, identical to the bearded capuchins investigated in the present study.

Notably, there are varying numbers of contrahens muscles across the species considered here: humans and gorillas present no such muscles; chimpanzees present two muscles; and baboons present three muscles. Therefore, P_ijk_ was ascribed the values of 0, 0, ½, and ¾ for humans, gorillas, chimpanzees, and baboons, respectively, for the purpose of the CAI calculation.

**FIGURE 2:**
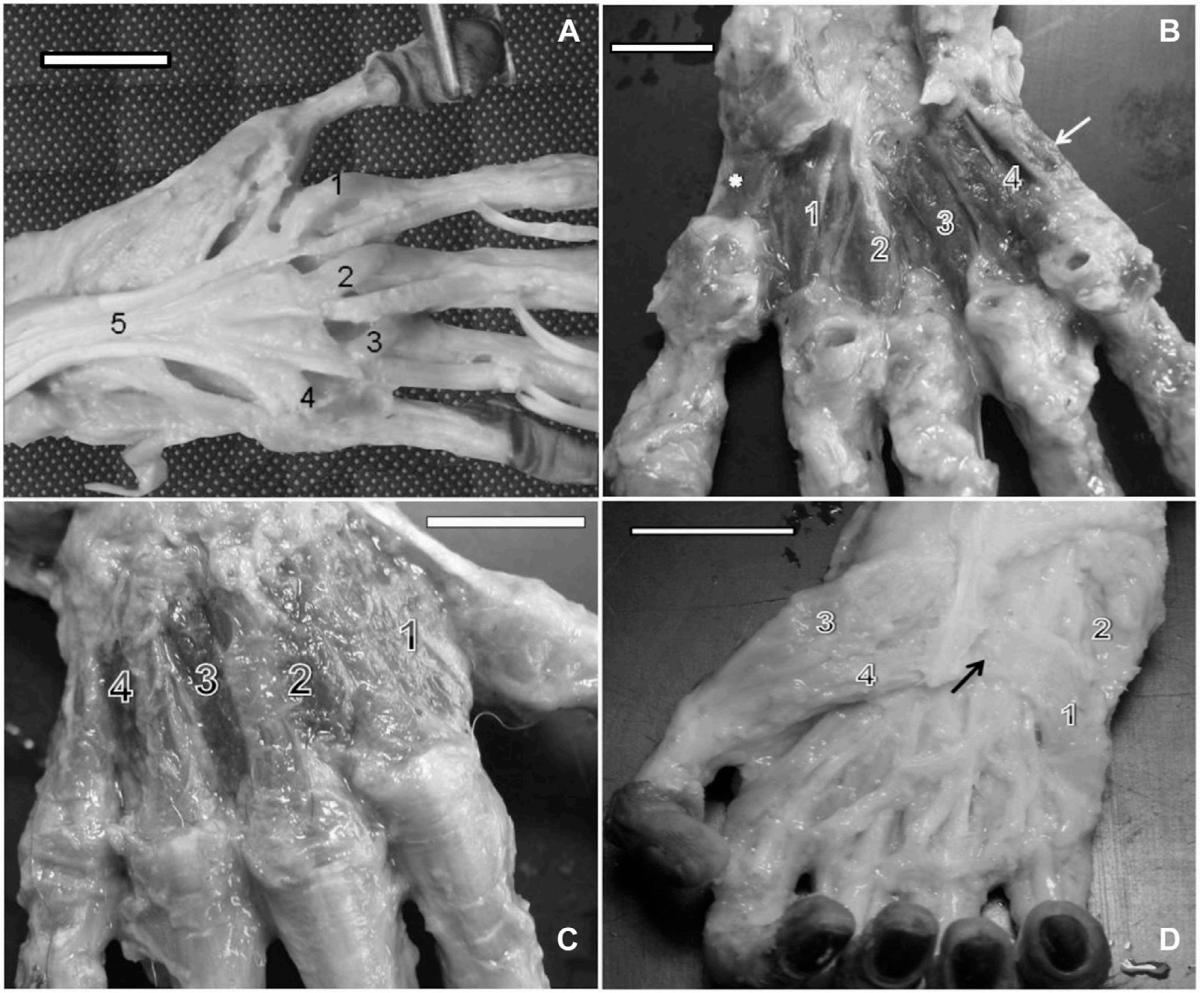
**(A)** Left hand of a bearded capuchin. Lumbrical muscles are numbered from 1 to 4. Number 5 indicates the tendon for the deep flexor muscle of the fingers (bar = 2.2 cm). **(B)** Left hand of a capuchin. Dissection revealed palmar interosseous muscles (1, 2, 3, and 4), the digit IV opposing muscle (arrow), and the thumb opposing muscle (*) (bar = 2.4 cm). **(C)** Right hand of a bearded capuchin. Dissection revealed dorsal interossei (1, 2, 3, and 4; bar = 3.2 cm). **(D)** Right hand of a capuchin. Dissection revealed the origin tendon of the flexor digiti minimi brevis (arrow) and its ventral portion (1), abductor digiti minimi (2), abductor pollicis brevis (3), and flexor pollicis brevis muscles (4) (bar = 2.8 cm).

The *lumbrical* group of muscles comprised four muscles elongated along the sagittal plane. They had a double origin, except the first, from the tendons of the flexor digitorum profundus muscle in all bearded capuchins analyzed in this study; they were inserted onto the extensor aponeurosis and onto the lateral side of the proximal phalanges of fingers II–V (Figure 2A). According to Swindler and Wood (1973), they are identical in all primates: the first and second lumbricals are innervated through the median nerve, while the third and fourth lumbricals are innervated through the ulnar nerve.^31^ In the bearded capuchins investigated in this study, this pattern of innervation was not observed in their four hands, while in the others, the branches were cut.

Gibbs (1999) reported that all modern humans and apes present four lumbricals (Standring, 2008), which originate from the tendon of the flexor digitorum profundus, as observed here in the bearded capuchins; however, the first lumbrical originates from the tendon of the flexor pollicis longus only in gorillas.

Moreover, there were four *palmar interosseous* muscles located between two adjacent metacarpal bones. The first palmar interosseous muscle originated in the lateral margin of the metacarpal II and the retinaculum of the flexor muscles and was inserted into the base of the proximal phalanx of digit II, next to the insertion tendon of the first dorsal interosseous muscle. The second palmar interosseous muscle originated in the medial margin of metacarpal II and in the retinaculum of the flexor muscles and was also inserted at the base of the proximal phalanx of digit II but next to the metacarpophalangeal articulation capsule. The third muscle stemmed from the medial margin of metacarpal IV and was inserted into the base of the proximal phalanx of digit IV, associated with the corresponding metacarpophalangeal articulation capsule. Finally, the fourth muscle originated in the medial margin of metacarpal V and was inserted at the base of the proximal phalanx of digit V (Figure 2B). All muscles were innervated through the ulnar nerve and supplied by branches of the ulnar artery.

The palmar interosseous muscles range from three to seven among primates. Baboons have seven, with different origins (i.e., with two heads for all of them), with the same insertions in relation to *Sapajus* (Swindler and Wood, 1973).

Gibbs (1999) reported five such muscles in gorillas and from six to seven in chimpanzees, and in modern humans, *Pan paniscus*, and gorillas, those muscles are innervated through the deep ramus of the ulnar nerve; in great apes and modern humans, they have the same origin and were inserted into the proximal phalanges and dorsal aponeurosis of each finger, identical to what was observed in *Sapajus*; however, in gorillas, they are inserted onto the capsules of the metacarpophalangeal joints.

*Dorsal interossei* are the only intrinsic muscles in the dorsal portion of the hand. In bearded capuchins, they originated on the adjacent sides of two metacarpal bones. They were all feather-shaped muscles except for the first, which was flabelliform. No insertions in digits I and V were observed. Digits II and III received insertions in the lateral and medial sides of their proximal phalanges from the first, second, and third interosseous muscles, whereas digit IV received an insertion from the fourth interosseous muscle in its medial portion (Figure 2C). They were innervated through the ulnar nerve in bearded capuchins.

This group is identical in all of the studied primates considered here (Swindler and Wood, 1973; Gibbs, 1999); however, an additional origin of the first dorsal interosseous from the pyramidal bone was reported in gorillas, while in chimpanzees, the fourth dorsal interosseous is inserted onto the lateral side of the metacarpal V,^31^ different from what was observed in bearded capuchins. All dorsal interossei are innervated through the ulnar nerve in all primates studied here (Swindler and Wood, 1973; Gibbs, 1999).

Lastly, the central muscle group of bearded capuchins is more similar to those in baboons and chimpanzees and exhibits more differences when compared with modern humans and gorillas. The main differences between bearded capuchins and baboons in central muscles were observed in the palmar interossei in terms of the origin and number (Table 3).

#### 3.2.2 Hypothenar muscles

The *palmaris brevis* in bearded capuchins exhibited a triangular form, originated from the palmar aponeurosis, and inserted onto the medial part of the hand skin (Figures 1A, B) (Table 3). Furthermore, it was innervated through the ulnar nerve. Data on the palmaris brevis muscle are scarce in primates, but it originates from the palmar fascia in modern humans and chimpanzees,^32^ from the annular ligament in modern humans and gorillas and also from the pisiform in gorillas; furthermore, it is inserted onto the skin of the ulnar border of the palm and is innervated through the ulnar nerve in all primates cited here (Swindler and Wood, 1973; Gibbs, 1999).

The palmaris brevis of bearded capuchins was identical to that of baboons, very similar to that of chimpanzees, and presented some differences from those of humans and gorillas in that order (Table 4).

**TABLE 4:** Hypothenar muscle of capuchins with the origins and insertions compared with modern humans, apes, and baboons.

Hypothenar muscle	Origin	Insertion	Human	Chimpanzee	Gorilla	Baboon
1. *Palmaris brevis*	Palmar aponeurosis	Medial part of the hand skin	Originates from the palmar aponeurosis and annular ligament. Identical insertion. CAI = 0.111	Identical to bearded capuchins. CAI = 0.056	Originates from the palmar aponeurosis, annular ligament, and pisiform. Identical insertion. CAI = 0.167	Identical to bearded capuchins. CAI = 0
2. *Abductor digiti minimi*	Pisiform, hamulus of the hamate, and medial portion of the flexor retinaculum	Digit V at the medial base of the proximal phalanx and metacarpophalangeal capsule	Does not present an origin from the hamulus of the hamate and has two additional origins. Identical insertion. CAI = 0.167	Does not present an origin from the hamulus of the hamate. One different insertion. CAI = 0.167	Does not present an origin from the hamulus of the hamate. One different insertion. Identical insertion. CAI = 0.111	Does not present an origin from the hamulus of the hamate. One different insertion. CAI = 0.111
3. *Flexor digiti minimi brevis*	Flexor retinaculum	Digit V at the base of the proximal phalange	Originates at the hamulus of the hamate and flexor retinaculum. Identical insertion. CAI = 0.111	Originates at the hamulus of the hamate and flexor retinaculum. Identical insertion. CAI = 0.111	Originates at the hamulus of the hamate and flexor retinaculum. Additional insertion onto the capsule of the metacarpophalangeal joint. CAI = 0.167	Originates at the hamulus of the hamate and flexor retinaculum. Identical insertion CAI = 0.056
4. *Opponens digiti minimi*	Hamulus of the hamate, pisiform bone, and flexor retinaculum	Medial margin at the distal end of metacarpal V and the metacarpophalangeal capsule	Does not present an origin from the pisiform. Identical insertion*.* CAI = 0.111	Does not present an origin from the pisiform. Identical insertion*.* CAI = 0.111	Does not present an origin from the pisiform. Identical insertion*.* CAI = 0.111	Does not present an origin from the pisiform. Identical insertion*.* CAI = 0.056
Hypothenar			GCAI = 0.125	GCAI = 0.111	GCAI = 0.139	GCAI = 0.056

**FIGURE 3:**
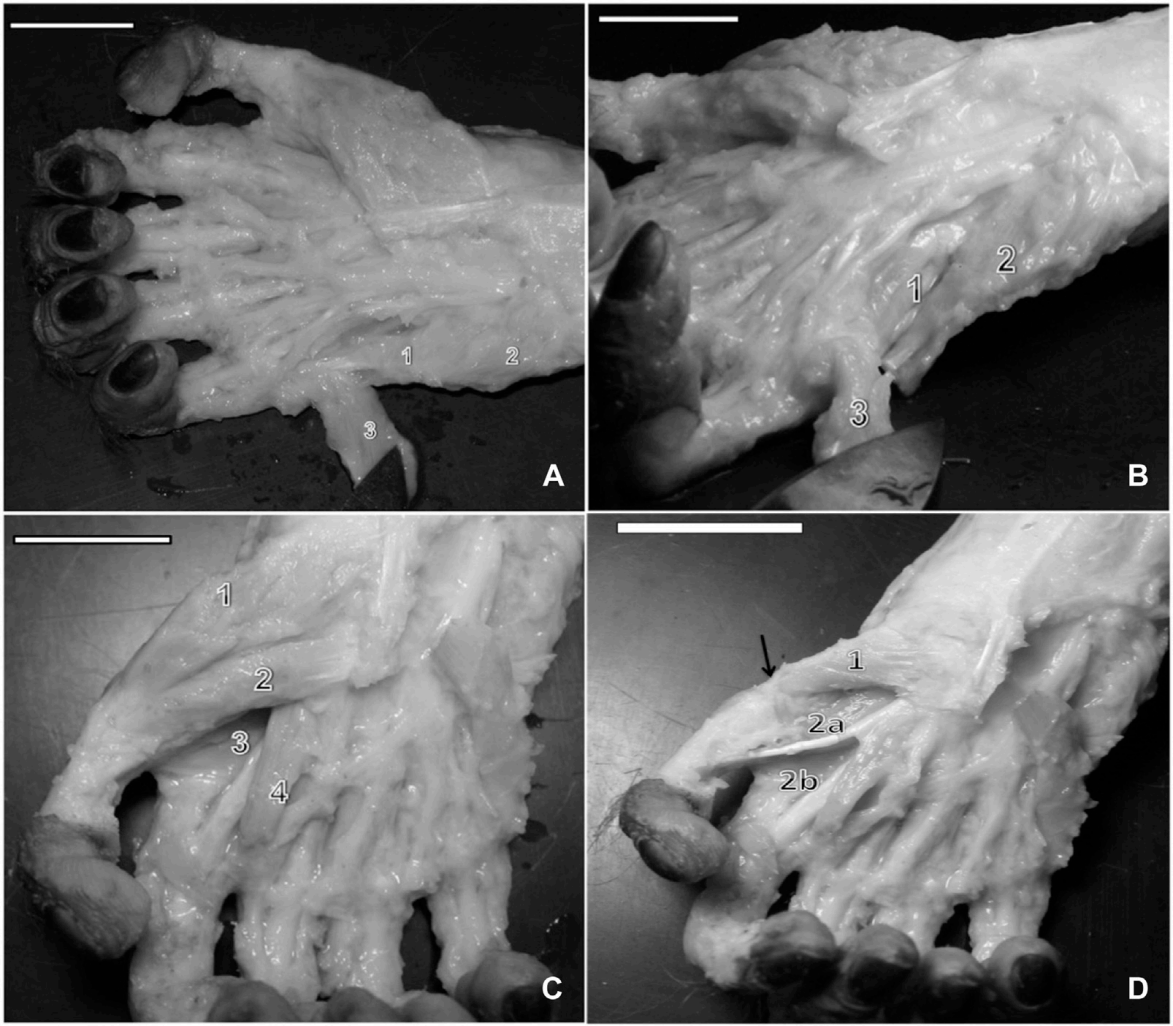
**(A)** Right hand of a capuchin. Numbers 1 and 2 indicate the venter of the abductor digiti minimi muscle, and number 3 indicates the center of the flexor digiti minimi brevis, opened (bar = 2.8 cm). **(B)** Right hand of a capuchin Dissection revealed the opponens digiti minimi (1), (2) abductor digiti minimi muscle, cut, and (3) flexor digiti minimi brevis, opened (bar = 2.8 cm). **(C)** Right hand of a capuchin. Dissection revealed digit I’s (1) abductor pollicis brevis, (2) flexor pollicis brevis; (3) adductor muscle, transverse head, and (4) the first lateral contracting muscle, cut (bar = 2.8 cm). **(D)** Right hand of a capuchin. Dissection revealed (arrow) the tendon of the insertion of (1) the opponens pollicis muscle and the adductor muscle and the (2a) oblique head and (2b) the transverse head (bar = 3.0 cm).

The *abductor digiti minimi* is located at the superficial medial portion of the hand, originated from the pisiform, hamulus of the hamate, and medial portion of the flexor retinaculum (Figures 2D, 3A, B). It is inserted into the medial portion of the metacarpophalangeal articulation capsule of digit V, extending to the base of the proximal phalanx blended with the flexor digiti minimi brevis (Figures 2D, 3A, B). It is innervated through the ulnar nerve.

In humans, chimpanzees, baboons (Swindler and Wood, 1973), and gorillas (Gibbs, 1999), the muscle originates from the pisiform bone and carpal ulnar ligament, although it originates from the pisohamate ligament and the tendon of the flexor carpi ulnaris in humans (Gibbs, 1999). None of the primates exhibited an origin from the hamulus of the hamate, as verified in *Sapajus*. The insertion for this muscle in these species is identical to that in bearded capuchins, namely, onto the medial base of the proximal phalanx^32^ and blended with the flexor digiti minimi brevis. As a variation in modern humans, however, it is also inserted onto the extensor aponeurosis of digit V in modern humans as well as in chimpanzees and onto the capsule of the metacarpophalangeal joint in gorillas (Gibbs, 1999), similar to bearded capuchins. In all primates studied here, innervation takes place through the ulnar nerve (Swindler and Wood, 1973; Gibbs, 1999).

According to the CAI calculation, the abductor digiti minimi was found to be similar among baboons, gorillas, and bearded capuchins.

In bearded capuchins, the *flexor digiti minimi brevis* muscle is a flat muscle originating from the flexor muscle retinaculum in the medial portion of the hand (Figures 2D, 3A, B). It lies obliquely and is inserted into the medial part of the digit V proximal phalanx base; it is distally located at the insertion of the abductor digiti minimi muscle, with which it shares its fibers, and is innervated through the ulnar nerve.

The origin at the hamate’s hamulus and flexors’ retinaculum, insertion onto the base of the proximal phalange of digit V, which is in common with the abductor digiti minimi, and innervation through the ulnar nerve have been reported for all other primates (Swindler and Wood, 1973; Gibbs, 1999). However, Gibbs (1999) reported additional insertions in gorillas onto the metacarpophalangeal joint and the extensor tendon on the dorsal surface. The flexor digiti minimi brevis is more similar to that of baboons, whereas the additional two insertions are more different from those of gorillas.

The *opponens digit minimi* is located beneath the abductor digiti minimi. In capuchins, it originates from the hamate’s hamulus, pisiform bone, and flexor muscle retinaculum. It is inserted onto the distal end of metacarpal V, at the medial margin, and in the articulation capsule of the metacarpophalangeal joint (Figures 3C, D).

In all other primates, it originates from the hamate bone and the flexor muscle retinaculum, the insertion occurs on the ulnar margin of metacarpal V, and innervation takes place through the ulnar nerve (Gibbs, 1999; Standring, 2008). The origin of the opponens digiti minimi in the pisiform in primates other than the *Sapajus* was not mentioned in any of the articles analyzed here, but other features of the opponens digiti minimi were identical among all primates.

The difference in the calculated CAI for baboons compared to *Sapajus* stems from the origin of the pisiform muscle in the latter. The variations in CAI for *Pan*, gorillas, and humans arise due to the presence of two palmaris arcus muscles in their hands. Specifically, the CAI values were 0.056 for baboons and 0.111 for the other three species (Table 3).

The hypothenar muscles in capuchins exhibit distinct origins compared with the other primates considered here, but differences exist in insertions, mainly in relation to gorillas. Notwithstanding, according to Swindler and Wood (1973), the abductor digiti minimi occasionally exhibits an origin in the hamate’s hamulus in humans. However, Standring (2008) reported a consistent origin in the pisohamate ligament for humans.

In the CAI calculation, the differences in bearded capuchins in terms of origins and insertions were largest compared to modern humans and gorillas.

#### 3.2.3 Thenar muscles

Swindler and Wood (1973) found that the *abductor pollicis brevis* muscle is the main component of the thenar eminence in chimpanzees, baboons, and humans (Table 5). It also seems to be the case in gorillas, although this was not directly mentioned by Gibbs (1999). In capuchins, it overlays digit V’s opponent muscle and the lateral part of the short flexor muscle. It originates from the palmar aponeurosis, flexor muscle retinaculum, and scaphoid bone and is inserted into the lateral portion of the proximal phalanx base, associated with the corresponding articulation capsule (Figures 3C, D).

**TABLE 5:** Thenar muscles of capuchins with the origins and insertions compared with modern humans, apes, and baboons.

Thenar muscle	Origin	Insertion	Human	Chimpanzee	Gorilla	Baboon
1. *Abductor pollicis brevis*	Palmar aponeurosis, flexor retinaculum, and scaphoid bone	Digit I at the lateral base of the proximal phalanx	Origin at trapezium and scaphoid^a^ and flexor retinaculum. Identical insertion. CAI = 0.111	Trapezium bone and flexor retinaculum. CAI = 0.111	Trapezium bone and flexor retinaculum. CAI = 0.111	Trapezium bone and flexor retinaculum. CAI = 0.056
2. *Flexor pollicis brevis*	Medial portion at the distal end of the palmar aponeurosis and the distal portion of the flexor retinaculum	Digit I at the antero-medial side of the proximal phalanx and articular capsule	Two heads that originate from the flexor retinaculum and the tuberculum of trapezium, trapezoid, and capitate. CAI = 0.222	Similar to humans. The ulnar head is present in 3/12. CAI = 0.180	Similar to humans. The ulnar head is present in 1/16. CAI = 0.087	Origin at the trapezoid and metacarpal II. CAI = 0.167
3. *Opponens pollicis*	Trapezium and trapezium–metacarpal I articular capsule	Anterior side of metacarpal I and the base of the metacarpophalangeal joint	Identical origin. Insertion at the lateral side of metacarpal I. CAI = 0.278	Similar to humans. CAI = 0.278	Similar to humans. CAI = 0.278	Similar to humans. CAI = 0.222
4. *Adductor pollicis*	Oblique head: metacarpal II diaphysis, capitate, and scaphoid. Transverse head: distal end of metacarpal III	Digit I at the medial portion of the base of the proximal phalanx	Origin at capitate and metacarpal II and III. Identical insertion. CAI = 0.167	Similar to humans. CAI = 0.167	Origin at the base of metacarpals I, II, and/or III, tendon of contrahentes, and metacarpophalangeal joints II and III. CAI = 0.222	Origin at the base of metacarpals II and III, tendon of contrahentes, and metacarpophalangeal joints II and III. CAI = 0.167
Thenar group			GCAI = 0.195	GCAI = 0.184	GCAI = 0.175	GCAI = 0.153

^a^ Referred to as “navicular” by Swindler and Wood (1973).

The *abductor pollicis brevis* was observed to originate from the scaphoid bone in all *Sapajus* specimens dissected in this study and was innervated through the ulnar nerve. This is without precedent in comparison with primate species. In humans, discrepancies exist regarding its origin from the navicular bone (Swindler and Wood, 1973) and scaphoid bone (Gibbs, 1999), but studies are in agreement regarding its origin from the trapezium bone. Napier (1955) indicated the flexor retinaculum, tuberculum of the scaphoid, and the crest of the trapezium to be the common origins for this muscle in humans and that it shares fibers with the flexor pollicis brevis. In gorillas and baboons, the bone origin starts from the trapezium bone (Swindler and Wood, 1973; Gibbs, 1999). Once again, insertion and innervation through the ulnar nerve are identical in all primates considered here; however, branches from the median nerve were cited for humans (Gibbs, 1999).

The *flexor pollicis brevis* originates from the medio-distal portion of the palmar aponeurosis and distal portion of the flexor muscle retinaculum; furthermore, it is inserted into the anterior-medial margin of the proximal phalanx, associated with the articulation capsule (Figures 3C, D). The deep head of the short flexor muscle was not observed in the bearded capuchin specimens. It shares fibers with the abductor pollicis brevis. The tendon of insertion of the long flexor muscle is located deep and medially relative to the distal portion of the flexor pollicis brevis. It is innervated through the median nerve.

In apes and baboons, the flexor pollicis brevis presents two heads, namely, the radial or superficial and the ulnar or deep head (Swindler and Wood, 1973; Gibbs, 1999). The radial head originates from the flexor retinaculum in modern humans, all apes (Gibbs, 1999), and baboons (Swindler and Wood, 1973) and from the tubercle of the trapezium in chimpanzees and modern humans (Swindler and Wood, 1973; Gibbs, 1999) and is innervated through the median nerve. The ulnar head originates from the trapezoid and capitate in modern humans and chimpanzees when present (Swindler and Wood, 1973; Gibbs, 1999; Standring, 2008) and from the trapezoid and base of metacarpal II in baboons, which is always present (Gibbs, 1999). Moreover, it is innervated through the ulnar nerve. Insertion is identical in all primates (Swindler and Wood, 1973; Gibbs, 1999).

Gibbs (1999) claimed that the ulnar head is present in 3/12 of *Pan* and 1/16 of gorillas; therefore, the frequency (P_ijk_) of innervation, origin, and the number for the calculation of CAI in each of these species was ½ (1–3/4 × 3/12) and ½ (1–3/4 × 1/16), respectively.

Due to doubts about the presence of the ulnar head in modern humans, Day and Napier (1963) analyzed the heads of the flexor pollicis brevis muscle in 65 human corpses. They concluded that it presents two heads at the origin, thus settling the controversy surrounding this issue.

According to Gibbs (1999), an ulnar head is found in all primates, in which the thumb performs true opponency. A detailed anatomical study (Aversi-Ferreira et al., 2014c) demonstrated that bearded capuchins do not perform opponency, despite their high cognitive capacity for tool use, but they use precision grip as a lateral pinch.

The flexor pollicis brevis is more similar between bearded capuchins and gorillas, mainly because of the similarity of the frequency of the number.

The *opponens pollicis* originates from the trapezium bone and the trapezium–metacarpal articulation capsule. It is inserted onto the anterior margin of metacarpal I and the base of the metacarpophalangeal capsule, and it is innervated through the median nerve. It is located underneath the abductor pollicis brevis, with which it shares fibers (Figure 3C).

In chimpanzees, baboons, and modern humans, the opponens pollicis is innervated through the median nerve, originating from the radial transverse carpal ligament and trapezium (Swindler and Wood, 1973). According to Gibbs (1999), it is absent in *Pan*. The insertion is similar to the primates studied here, namely, onto the radial margin of metacarpal I; however, in bearded capuchins, the position of the insertion is anteriorly placed, which is an important aspect in relation to the angle of the movement of the pollicis. In *Sapajus*, it is more similar to baboons because of the supplying pattern.

This muscle exists in all primates, with its development peaking in humans according to Swindler and Wood (1973). Napier (1955) defined it as a rotatory muscle located in a privileged position for acting on the trapezium–metacarpal articulation. The different insertions found in capuchins indicate more of a flexing rather than a rotating action (Aversi-Ferreira et al., 2014c). It does not allow, for instance, the cushion of the thumb distal phalanx to rotate toward the cushion of the other digits. In fact, the flexing action of digit I’s opponent muscle is more consistent with the phylogenetic history since in lower mammalians, it is fused with the flexor pollicis brevis in digit I.

The *adductor pollicis* of capuchins has two heads: oblique and transverse. The transverse head originates from the distal end of the third metacarpal and is inserted into the medial portion of the proximal phalanx base of digit I (Figures 3C, D). The oblique head has an identical insertion but a different origin, originating from metacarpal II’s diaphysis, capitate, and scaphoid bones. This last origin was not reported in the compared primates. The adductor pollicis is innervated through the ulnar nerve in bearded capuchins.

Moreover, humans and chimpanzees exhibit an identical origin for this head in the capitate bone and bases of metacarpal II and III; however, in baboons (Swindler and Wood, 1973) and gorillas (Gibbs, 1999), the origins from the capitate and scaphoid bones have not been reported. There are four origins for the two muscle heads in bearded capuchins with common insertion in three instances (namely, metacarpal II, capitate bone, and metacarpal III for the transverse head) in chimpanzees and modern humans; by contrast, in gorillas and baboons, they present five insertions (Swindler and Wood, 1973; Gibbs, 1999), but only two of them are identical in bearded capuchins. The insertion is identical in bearded capuchins and other primates, namely, onto the base of the first pollical phalanx, and the tendon of the distal phalanx has been reported in baboons, chimpanzees (Swindler and Wood, 1973), and gorillas (Gibbs, 1999), but it was not observed in *Sapajus*. For the primates studied here, the innervation of the adductor pollicis takes place through the ulnar nerve. Taken together, the capuchin’s thenar group is more similar to that of baboons.

According to the descriptive analysis and GCAI results (Tables 2, 3, 5), *Sapajus* hand muscles are similar to those of baboons. It is crucial to note that the vascularization of the hand is identical between capuchins and baboons but is different compared with the other primates (Aversi-Ferreira et al., 2007a; Aversi-Ferreira et al., 2007b; Aversi-Ferreira et al., 2007c; Aversi-Ferreira, 2009; Abreu et al., 2021; Aversi-Ferreira et al., 2021b), while innervation is identical in all of them (Marin et al., 2009), except for the presence of the second head of the flexor pollicis brevis, which is innervated through the ulnar nerve. A higher similarity with baboons was also reported for other parts of the thoracic limb, as cited in the Introduction section.

In addition, the thenar muscles in the human hand are stronger and bulkier than those in other primates (Young, 2003), even chimpanzees (Ogihara et al., 2005), whose hands are used as an evolutionary model for hominid tool use. Bearded capuchin thenar muscles are not as strong and bulky as those of chimpanzees and do not seem to be more adapted for tool use than those of baboons in terms of the muscular structure, corroborating the data of Aversi-Ferreira and colleagues (2014a).

**FIGURE 4:**
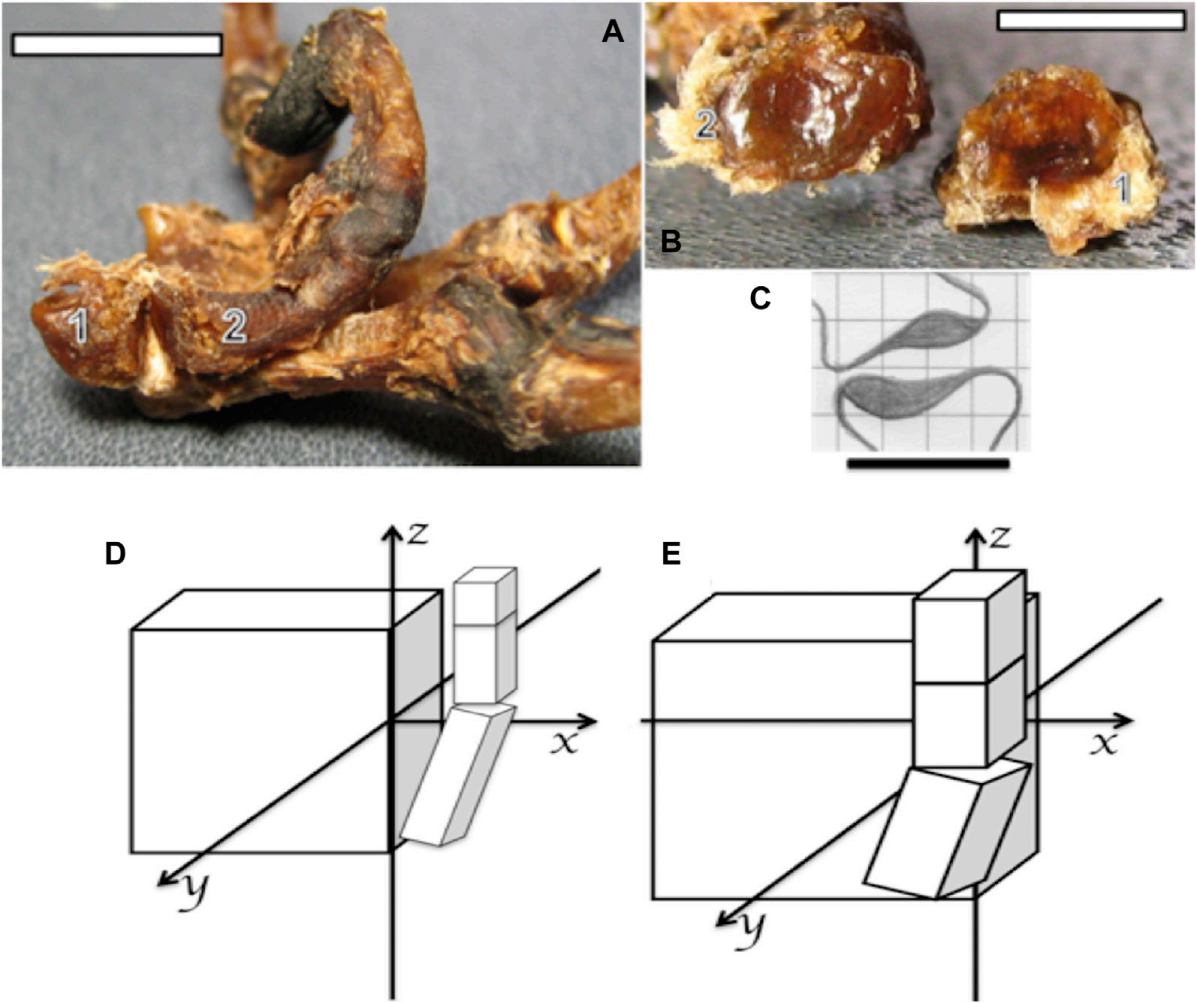
**(A,B)** Photographs of the bones and ligaments of the right hand of a capuchin. Number 1 indicates the trapezium and number 2 the metacarpal I. **(A)** Position of bones 1 and 2 in the formation of the trapezium–metacarpal joint (bar = 16 cm). **(B)** Articular faces of bones 1 and 2 showing concave (bone 2) and convex (bone 1) surfaces (bar = 32 cm). **(C)** Schematic association between the trapezium (down) and metacarpal I (up) to form a saddle joint, proportionally obtained from bearded capuchin bones (bar = 25 cm). **(D)** Schematic representation of a *Sapajus* hand, in which the projection of the thumb over the plane of the hand is limited to the *z*-axis; that is, the carpus aligns in a flat plate or a few centimeters past it in the latero-medial direction. **(E)** Schematic representation of a human hand, indicating the projection of the thumb over the plane of the hand; that is, the curved carpus and crossing parallel to the *z*-axis due to the anterior position of the trapezium bone (based on Aversi-Ferreira et al., 2014a; Aversi-Ferreira et al., 2014c).

According to Young (2003), the evolution of the human hand is associated with tool use and bipedalism, as bipedalism was necessary to permit the free use of their hands. Tocheri and colleagues (2008) suggested a correspondence between cognitive evolution and hand morphology in primates. Our analysis of *Sapajus* hand muscles does not support tool-use behavior in the way that it is credited to this genus. The hand of the *Sapajus* monkey is more similar to that of baboons in terms of the central, hypothenar, and thenar muscles as well as other structures observed here (Tables 2, 3, 5; Figure 4), while baboons do not exhibit the muscle requirements for the hand skills associated with fine tool use; nevertheless, capuchins perform tool use and demonstrate high cognition abilities (Lacreuse and Fragazy, 1997; Panger, 1998; Fragazy et al., 2004; Abreu et al., 2021). Other data regarding *Sapajus* hands have also demonstrated a primitive characteristic compared with great apes and modern humans (Aversi-Ferreira et al., 2010; Aversi-Ferreira et al., 2011; Aversi-Ferreira et al., 2011b; Aversi-Ferreira et al., 2014c).

The muscle anatomical data from this work expands upon previous bone studies of a *Sapajus* hand (Aversi-Ferreira et al., 2014c). Those studies demonstrated, through general measures of the carpus and digits, that these primates lack the capacity for true thumb opponency. Our findings further support this conclusion by revealing that the *Sapajus* carpus is straighter than the human carpus due to the absence of a central bone in the former. Consequently, the first metacarpus originates in the same line as the carpus plane in capuchins, hindering the movement of the cushion of the pollicis toward the base of the first phalanx of other digits. In fact, the opponens pollicis action observed in this work more closely resembles the flexor action rather than true opponency. This, along with the saddle trapezium–metacarpal joint observed in *Sapajus* (Aversi-Ferreira et al., 2014c), confirms that capuchins lack the capacity for human-like thumb opponency.

In contrast, while great apes possess the ability for thumb opponency, their thumbs are generally small and weak, limiting their object manipulation skills. Notwithstanding, despite lacking true opponency, *Sapajus* exhibits remarkable skills in manipulating objects (Fragaszy et al., 2004; Fragaszy et al., 2004). This dexterity relies on the combined strength of various hand movements, primarily involving the second and third digits, the first digit in conjunction with the second metacarpal/second phalanx base (Aversi-Ferreira et al., 2014c). Therefore, bearded capuchins leverage powerful hand muscles to achieve great hand skills in manipulating objects, deviating from the expectations set by Darwin’s theory of human evolution (Darwin, 1871). The combined insights into linear hand measures, bone anatomical analyses (Aversi-Ferreira et al., 2014c), and the data from this present work unveil an alternative pathway for achieving advanced hand manipulation in *Sapajus*, distinct from both great apes and modern humans.

## 4 Final considerations

Considering other works on hand manipulation and anatomy, the *Sapajus* trapeziometacarpal joint exhibits a latero-laterally concave part (metacarpal I) and latero-laterally convex part (trapezium), generating a saddle joint-like arrangement (Aversi-Ferreira et al., 2014a). This differs significantly from the configuration observed in great apes and modern humans (Figures 4A–C).

Such a joint restricts the medial rotation of thumbs, hindering true opponency (Napier, 1961; Napier, 1980), as shown in Figure 4. Aversi-Ferreira et al. (2006) reported that capuchins possess nine carpal bones, one more than adult great apes and humans. This central bone contributes to a flat carpal plane, indicating a relatively primitive hand structure. Conversely, great apes and modern humans exhibit a curved carpus capable of true opponency (Aversi-Ferreira et al., 2011). Therefore, the capuchin hand pattern positions the thumb closer to the carpal plane, limiting opponency, while the curved human carpus allows the thumb to move above the hand plane and across the additional axes, namely, the *y-* and *x*-axes, and parallel to the *z*-axis, as indicated in Figures 4D, E.

These analyses reveal that the *Sapajus* hand more closely resemble those of arboreal primates (Aversi-Ferreira et al., 2014a), consistent with the findings of the current study and other studies comparing the primate blood supply (Aversi-Ferreira et al., 2020; Aversi-Ferreira et al., 2021b). However, linear hand proportion measurements suggest that capuchin hands are closer to those of humans than other primates (Almecíja et al., 2015). Despite that, a three-dimensional analysis (Aversi-Ferreira et al., 2014c) demonstrated that the flat plane hand of *Sapajus* is more similar to baboons than humans.

Through precise analyses of anatomical structures rather than behavioral observations, we derived propositions for generating a deductive corollary regarding the tool use and high manual abilities of capuchins. Subsequently, we employed inductive reasoning to generate new hypotheses for subsequent works exploring thumb opponency, tool use associated with encephalon characteristics (Borges et al., 2015; Aversi-Ferreira et al., 2021a; Abreu et al., 2021), and primate evolution.

According to our comparative analysis of hand muscles, the bearded capuchin hand exhibits a closer similarity to that of baboons than modern humans and great apes based on both the CAI calculations and gross anatomical observations. This is primarily due to the absence of the ulnar head of the flexor pollicis brevis and the flexion action of the opponens pollicis, which are the feature characteristics of primates lacking true opponency (Gibbs, 1999). We propose this primitive characteristic of bearded capuchin hand muscles as **proposition 1**.

Furthermore, Aversi-Ferreira et al. (2014c) concluded that capuchins possess a relatively primitive hand based on the articular surfaces of the carpus–thumb joint and carpal dimensions, indicating limited thumb movement. This finding reinforces the distance between capuchins and humans/great apes and suggests a greater affinity with arboreal specialist primate hands. However, in terms of hand proportions, using the intrinsic hand proportion method (Almécija et al., 2015), *Sapajus* was found to be closer to modern humans due to its relative thumb length. Notably, Almécija et al.’s (2015) analysis was linear, while Aversi-Ferreira et al. (2014c) considered the three-dimensional shape of the carpal bones, including the features of the hand palm (i.e., less arched carpus and less tridimensionality compared to apes and humans). Therefore, based on both our muscle data and the bone structures described by Aversi-Ferreira et al. (2014c), we propose that the bearded capuchin hand exhibits primitive characteristics, constituting **proposition 2**.

Indeed, the corollary derived from propositions 1 and 2 suggests that the *Sapajus* hand lacks the morphological characteristics necessary for enabling true thumb opponency or advanced tool use. This makes it a relative primitive hand among primates, supported by the analyses of both bones (Aversi-Ferreira et al., 2014c) and muscles in this work. This conclusion is further corroborated by the evolutionary distance between *Sapajus* and humans.

The association between thumb opponency, tool use, and high cognitive skills in primates is well established, supported by various studies focusing on apes (Napier, 1952; Napier, 1961; Ogihara et al., 2005; Tocheri et al., 2008). Given the high cognitive and manipulative abilities of capuchins, exploring their evolutionary path toward these characteristics becomes intriguing (Bortoff and Strick, 1993). The standard evolutionary model for these skills in apes emphasizes the pivotal role of adaptation to bipedalism and tool use (Young, 2003; Tocheri et al., 2008). While bearded capuchins exhibit only occasional and limited bipedalism (Demes, 2011), making free hand movements challenging during locomotion, they utilize their hands freely during intermittent bipedal phases, such as when foraging. Phylogenetic studies based on DNA analysis indicated that the *Sapajus* genus is relatively distant from apes (Goodman et al., 1998). Nevertheless, bearded capuchins present cognitive abilities that parallel those of great apes (Reader et al., 2011). In light of their thoracic limb morphology (Aversi-Ferreira et al., 2005; Aversi-Ferreira et al., 2010; Aversi-Ferreira et al., 2014c), especially the form of their hand, capuchins seem to have undergone a divergent evolutionary process to achieve manipulation, indicating that behavioral convergence did not follow anatomical convergence with the apes.

It has recently been argued that the adoption of incremental terrestriality by arboreal primate species may have been crucial to the development of tool use in primates (van Schaik et al., 1999). This account is not necessarily in opposition to ours. First, terrestrialism does not precisely equate to bipedalism in terms of the conditions for complex manipulation. Although bipedalism substantially spares the upper limbs from the locomotory function, the use of complex tools is generally a stationary activity. Furthermore, terrestriality without bipedalism could still be advantageous for arboreal species in the handling of objects since less effort, and consequently, fewer limbs are necessary for maintaining balance on the ground. Second, reports exist on complex tool use in the arboreal habitats of wild capuchins and chimpanzees (Sugiyama, 1997; Boesch et al., 2009; Sanz and Morgan, 2009; Souto et al., 2011). This suggests that arborealism does not prevent the complex manipulation of objects completely, even if terrestriality seems to favor more complex interactions with tools (Meulman et al., 2012). Regardless, the present findings support the hypothesis that refined manipulatory skills may emerge in the absence of erect bipedalism.

According to Darwin (1871) and Washburn (1963), bipedalism, large brains, manipulative hands, tool use, and language are the interconnected traits associated with progressive hominid evolution.

The present investigation agrees with the view expressed by Fleagle (1988) that “the cluster of features characterizing living humans are not necessarily linked but are rather evolved one by one.” We have demonstrated that the morphological basis of the manipulatory behavior in *Sapajus* sp. is based on different hand traits (especially in the thenar sector) than those found in catarrhines (selected Old World monkeys, great apes, and Hominidae). In the latter infraorder, precision grips facilitated by a hypermobile trapezium–metacarpal joint permitted fine manipulation, leading to tool use and manufacture; that is, *Sapajus* sp. does not present this model. It uses most of its pliable carpal and metacarpal joints to “fold in” the palm and fingers around the object to be manipulated. Several Old and New World primates indicate that a habitual arboreal locomotory behavior provides an alternative pathway for manipulatory behavior and neocortical growth (Abreu et al., 2021) as well as elaboration from the one demonstrated by advanced hominids. Further studies employing detailed analyses of hand bone proportions using dimensional analysis would be valuable in further elucidating the evolutionary trajectory of the primate hand morphology and its relationship with manipulative and cognitive abilities. In summary, the morphological and phylogenetical findings derived from DNA analyses place capuchins as distantly related to apes. Therefore, the obligatory relationship between erect bipedalism, manipulatory hands centered on the hypermobile trapezium–metacarpal joint, elaboration of the neocortex leading to tool use/manufacture, and language that requires fresh scrutiny. Overall, convergent encephalization indices and cognitive capacities seem to allow capuchins to use their divergent and relatively limited morphological hand apparatus to a high degree of skill.

## Data Availability

All relevant data is contained within the article. The source specimens for the measured data are available at the University of Tocantins, Brazil. Further inquiries can be directed to the corresponding author.

## References

[B1] Abreu T. Tavares M. C. Vieira R. B. Rodrigues R. Pissinati A. Aversi-Ferreira T. A. (2021). Comparative anatomy of the encephalon of new world primates with emphasis for the *Sapajus* sp. PLoS One 16, e0256309. 10.1371/journal.pone.0256309 34469439 PMC8409804

[B2] Almécija S. Smaers J. Jungers W. (2015). The evolution of human and ape hand proportions. Nat. Commun. 6, 7717. 10.1038/ncomms8717 26171589 PMC4510966

[B3] Aversi-Ferreira R. A. Nishijo H. Aversi-Ferreira T. A (2015) Reexamination of statistical methods for comparative anatomy: examples of its application and comparisons with other parametric and nonparametric statistics. Biomed. Res. Int 2015;:902534. 10.1155/2015/902534 26413553 PMC4564798

[B4] Aversi-Ferreira RAGMF Abreu T. Pfrimer G. A. Silva S. F. Ziermann J. M. Carneiro-e-Silva F. O. (2013) Comparative anatomy of the hind limb vessels of the bearded capuchins (*Sapajus libidinosus*) with apes, baboons, and *Cebus capucinus*: with comments on the vessels' role in bipedalism. BioMed Res. Int 2013:737358. 10.1155/2013/737358 24396829 PMC3874347

[B5] Aversi-Ferreira RAGMF Bretas R. V. Maior R. S. Davaasuren M. Paraguassú-Chaves C. A. Nishijo H. (2014a) Morphometric and statistical analysis of the palmaris longus muscle in human and non-human primates. BioMed Res. Int 2014:178906–6. 10.1155/2014/178906 24860810 PMC4016873

[B6] Aversi-Ferreira RAGMF Maior S. R. Aziz A. Ziermann J. M. Nishijo H. Tomaz C. (2014c). Anatomical analysis of thumb opponency movement in the capuchin monkey (*Sapajus* sp). PLos One 9 (2), e87288. 10.1371/journal.pone.0087288 24498307 PMC3911977

[B7] Aversi-Ferreira RAGMF Marin K. A. Silva F. O. C. Aversi-Ferreira T. A. (2011). Comparative anatomy of the thigh nerve of *Cebus libidinosus* (Rylands et al., 2000). Braz. J. Veterinary Res. 31, 261–266. 10.1590/S0100-736X2011000300013

[B8] Aversi-Ferreira RAGMF Vieira S. V. Tomaz C. Aversi-Ferreira T. A. (2014b). Comparative anatomy of the pelvic vessels in the bearded capuchin (*Sapajus libidinosus*) and baboons, apes and modern humans. Folia Primatol. (Basel) 85 (4), 252–264. 10.1159/000366061 25377625

[B9] Aversi-Ferreira T. A. (2009). Comparative anatomical description of forearm and hand arteries of *Cebus libidinosus* . Int. J. Morphol. 27 (1), 219–226. 10.4067/S0717-95022009000100037

[B10] Aversi-Ferreira T. A. Aversi-Ferreira RAGMF Bretas R. V. Nishimura H. Nishijo H. (2016). Comparative anatomy of the arm muscles of the Japanese monkey (*Macaca fuscata*) with some comments on locomotor mechanics and behavior. J. Med. Primatol. 45 (4), 165–179. 10.1111/jmp.12222 27297259

[B11] Aversi-Ferreira T. A. Borges K. M. Goncalves-Mendes M. T. Caixeta L. F. (2021a). Gross anatomy of the longitudinal fascicle of *Sapajus* sp. PLoS One 16 (6), e0252178. 10.1371/journal.pone.0252178 34166386 PMC8224874

[B12] Aversi-Ferreira T. A. Diogo R. Potau J. M. Bello G. Pastor J. F. Aziz M. A. (2010). ComparativeAnatomical Study of the Forearm Extensor Muscles of *Cebus libidinosus* (Rylands et al., 2000; Primates, Cebidae), Modern Humans, and Other Primates, With Comments on Primate Evolution, Phylogeny, and Manipulatory Behavior. Anatomical Rec. 293 (12), 2056–2070. 10.1002/ar.21275 21082733

[B13] Aversi-Ferreira T. A. Freitas-Ferreira E. Aversi-Ferreira H. (2021b). Differences among the forelimb arteries in groups of primates and a mathematical model explanation. J. Med. Primatol. 50 (1), 21–28. 10.1111/jmp.12498 33063350

[B14] Aversi-Ferreira T. A. Freitas-Ferreira E. Aversi-Ferreira H. Cordeiro-de-Oliveira K. Lopes-de-Freitas G. Trevisan K. (2020). Comparative gross anatomy of the forelimb arteries of the Japanese monkey (*Macaca fuscata*) and a comparative pattern of forelimb arterial distribution in primates. BioMed Res. Int. 2020, 8635917–8636016. 10.1155/2020/8635917 32724814 PMC7381946

[B15] Aversi-Ferreira T. A. Lima-e-Silva M. S. Pereira-de-Paula J. Gouvea-e-Silva L. F. Penha-Silva N. (2005). Anatomia comparativa dos nervos do braço de *Cebus apella*. Descrição do músculo dorso epitroclear. Acta Sci. Biol. Sci. 27 (3), 291–296. 10.4025/actascibiolsci.v27i3.1338

[B16] Aversi-Ferreira T. A. Maior R. S. Carneiro-E-Silva F. O. Aversi-Ferreira RAGMF Tavares M. C. Nishijo H. (2011). Comparative anatomical analyses of the forearm muscles of *Cebus libidinosus* (Rylands et al. 2000): manipulatory behavior and tool use. PloS One 6 (7), e22165. 10.1371/journal.pone.0022165 21789230 PMC3137621

[B17] Aversi-Ferreira T. A. Pereira-de-Paula J. Lima-e-Silva M. S. Prado Y. C. L. Silva Z. (2007b). Estudo anatômico das artérias do ombro de *Cebus libidinosus* (RYLANDS, 2000; PRIMATES – CEBIDAE). Ciência Anim. Bras. 8 (2), 273–284. 10.5216/cab.v8i2.1352

[B18] Aversi-Ferreira T. A. Pereira-de-Paula J. Lima-e-Silva M. S. Silva Z. (2007a). Anatomy of the arteries of the arm of *Cebus libidinosus* (Rylands et al., 2000) monkeys. Acta Sci. Biol. Sci. 29 (3), 247–254. 10.4025/actascibiolsci.v29i3.473

[B19] Aversi-Ferreira T. A. Pereira-de-Paula J. Prado Y. C. L. Lima-e-Silva M. S. Mata J. R. (2007c). Anatomy of the shoulder and arm muscles of *Cebus libidinosus* . Braz. J. Morphol. Sci. 24 (2), 63–74.

[B20] Aversi-Ferreira T. A. Vieira L. G. Pires R. M. Silva Z. Penha-Silva N. (2006). Estudo anatômico dos músculos flexores superficiais do antebraço no macaco *Cebus apella* . Biosci. J. 22 (1), 139–144.

[B21] Boesch C. Head J. Robbins M. M. (2009). Complex tool sets for honey extraction among chimpanzees in Loango National Park, Gabon. J. Hum. Evol. 56 (6), 560–569. 10.1016/j.jhevol.2009.04.001 19457542

[B22] Borges K. C. Nishijo H. Aversi-Ferreira T. A. Ferreira J. R. Caixeta L. F (2015) Anatomical study of intrahemispheric association fibers in the brains of capuchin monkeys (*Sapajus* sp.). BioMed Res. Int 2015, 648128. 10.1155/2015/648128 26693488 PMC4676999

[B23] Bortoff G. A. Strick P. L. (1993). Corticospinal terminations in two new-world primates: further evidence that corticomotoneuronal connections provide part of the neural substrate for manual dexterity. J. Neurosci. 13 (12), 5105–5118. 10.1523/JNEUROSCI.13-12-05105.1993 7504721 PMC6576412

[B24] Canale G. R. Guidorizzi C. E. Kierulff M. C. Gatto C. A. (2009). First record of tool use by wild populations of the yellow-breasted capuchin monkey (*Cebus xanthosternos*) and new records for the bearded capuchin (*Cebus libidinosus*). Am. J. Primatology 71 (5), 366–372. 10.1002/ajp.20648 19206141

[B25] Darwin C. R. (1871). The descent of man, and selection in relation to sex. London: John Murray.

[B26] Day M. H. Napier J. R. (1963). The functional significance of the deep head of flexor pollicis brevis in primates. Folia Primatol. 1 (2), 122–134. 10.1159/000165787

[B27] Demes B. (2011). Three-dimensional kinematics of capuchin monkey bipedalism. Am. J. Phys. Anthropol. 145 (1), 147–155. 10.1002/ajpa.21484 21365612

[B28] Fleagle J. G. (1988). Primate adaptation and evolution. New York: Academic Press, Inc.

[B29] Fragaszy D. M. Izar P. Visalberghi E. Ottoni E. B. De Oliveira M. G. (2004). Wild capuchin monkeys (*Cebus libidinosus*) use anvils and stone pounding tools. Am. J. Primatology 64 (4), 359–366. 10.1002/ajp.20085 15580579

[B30] Fragaszy D. M. Visalberghi E. Fedigan L. M. (2004). The Complete Capuchin: the biology of the genus Cebus. Cambridge: Cambridge University Press.

[B31] Gibbs S. (1999). Comparative soft tissue morphology of the extant hominoidea, including man. Liverpool: University of Liverpool Press.

[B32] Goodman M. Porter C. A. Czelusniak J. Page S. L. Schneider H. Shoshani J. (1998). Toward a phylogenetic classification of primates based on DNA evidence complemented by fossil evidence. Mol. Phylogenetics Evol. 9 (3), 585–598. 10.1006/mpev.1998.0495 9668008

[B33] Hakeem A. Sandoval R. G. Jones M. Allman J. (1996). “Brain and life span in primates,” in Handbook of the psychology of aging Editors Birren J. E. Schaie K. W. ed 4th. (San Diego: Academic Press), 78–104.

[B34] Judge D. S. Carey J. R. (2000). Postreproductive life predicted by primate patterns. Journals Gerontology Ser. a-Biological Sci. Med. Sci. 55 (4), B201–B209. 10.1093/gerona/55.4.b201 10811147

[B35] Lacreuse A. Fragaszy D. (1997). Manual exploratory procedures and asymmetries for a haptic search task: a comparison between capuchin monkeys (*Cebus apella*) and humans. Laterality 2 (3-4), 247–266. 10.1080/713754275 15513067

[B36] Mannu M. Ottoni E. B. (2009). The enhanced tool-kit of two groups of wild bearded capuchin monkeys in the Caatinga: tool making, associative use, and secondary tools. Am. J. Primatology 71 (3), 242–251. 10.1002/ajp.20642 19051323

[B37] Marin K. A. Carneiro-e-Silva F. O. Carvalho A. V. Nascimento G. N. L. Prado Y. C. L. Aversi-Ferreira T. A. (2009). Anatomy of the nervous of forearm and hand of *Cebus libidinosus* (Rylands, 2000). Int. J. Morphol. 27 (3), 635–642. 10.4067/S0717-95022009000300003

[B38] Meulman E. J. M. Sanz C. M. Visalberghi E. van Schaik C. P. (2012). The role of terrestriality in promoting primate technology. Evol. Anthropol. 21 (2), 58–68. 10.1002/evan.21304 22499440

[B39] Napier J. R. (1952). The attachments and function of the abductor pollicis brevis. J. Anat. 86 (4), 335–341. 12999637 PMC1273687

[B40] Napier J. R. (1955). The form and function of the carpo-metacarpal joint of the thumb. J. Anat. 89 (3), 362–369. 13251966 PMC1244764

[B41] Napier J. R. (1961).Prehensibility and opposability in the hands of primates. Symposia of the Zoological Society of London, 5, 115–132.

[B42] Napier J. R. (1980). Hands. Princeton: Princeton University Press.

[B43] Ogihara N. Kunai T. Masato N. (2005). Muscle dimensions in the chimpanzee hand. Primates 46 (4), 275–280. 10.1007/s10329-005-0136-x 16143821

[B44] Panger M. A. (1998). Object-use in free-ranging white-faced capuchins (*Cebus capucinus*) in Costa Rica. Am. J. Phys. Anthropol. 106 (3), 311–321. 10.1002/(SICI)1096-8644 9696147

[B45] Pereira-de-Paula J. Prado Y. C. L. Tomaz C. Aversi-Ferreira T. A. (2010). Anatomical study of the main sulci and gyri of the *Cebus libidinosus* brain (rylands). Neurobiologia 73 (2), 65–78.

[B46] Reader S. M. Hager Y. Laland K. N. (2011). The evolution of primate general and cultural intelligence. Philosofical Trans. R. Soc. 366, 1017–1027. 10.1098/rstb.2010.0342 PMC3049098 21357224

[B47] Rilling J. K. Insel T. R. (1999). The primate neocortex in comparative perspective using magnetic resonance imaging. J. Hum. Evol. 37, 191–223. 10.1006/jhev.1999.0313 10444351

[B48] Sanz C. M. Morgan D. B. (2009). Flexible and persistent tool-using strategies in honey-gathering by wild chimpanzees. Int. J. Primatology 30, 411–427. 10.1007/s10764-009-9350-5

[B49] Souto A. Bione C. B. C. Bastos M. Bezerra B. M. Fragaszy D. Schiel N. (2011). Critically endangered blonde capuchins fish for termites and use new techniques to accomplish the task. Biol. Lett. 7, 532–535. 10.1098/rsbl.2011.0034 21389018 PMC3130233

[B50] Spagnoletti N. Izar P. Visalberghi E. (2009). Tool use and terrestriality in wild bearded capuchin monkey (*Cebus libidinosus*). Folia Primatol. 80, 142.

[B51] Spinozzi G. Truppa V. Lagana T. (2004). Grasping behavior in tufted capuchin monkeys (*Cebus apella*): grip types and manual laterality for picking up a small food item. Am. J. Phys. Anthropol. 125, 30–41. 10.1002/ajpa.10362 15293329

[B52] Standring S. (2008). Gray’s anatomy: the anatomical basis of clinical practice. London: Churchill Livingstone.

[B53] Stephan H. Andy O. J. (1969). Quantitative comparative neuroanatomy of primates - an attempt at a phylogenetic interpretation. Ann. N. Y. Acad. Sci. 167, 370–387. 10.1111/j.1749-6632.1969.tb20457.x

[B54] Sugiyama Y. (1997) Social tradition and the use of tool-composites by wild chimpanzees. Evol. Anthropol. 6:23–27. 10.1002/(sici)1520-6505(1997)6:1<23::aid-evan7>3.0.co;2-x

[B55] Swindler D. R. Wood C. D. (1973). An atlas of primates gross anatomy. Washington: University of Washington Press.

[B56] Tocheri M. W. Orr C. M. Jacofsky M. C. Marzke M. W. (2008). The evolutionary history of the hominin hand since the last common ancestor of *Pan* and *Homo* . J. Anat. 212, 544–562. 10.1111/j.1469-7580.2008.00865.x 18380869 PMC2409097

[B57] van Schaik C. P. Deaner R. O. Merrill M. Y. (1999). The conditions for tool use in primates: implications for the evolution of material culture. J. Hum. Evol. 36, 719–741. 10.1006/jhev.1999.0304 10330335

[B58] Washburn S. L. (1963). Classification and human evolution. Chicago: Aldine.

[B59] Young R. W. (2003). Evolution of the human hand: the role of throwing and clubbing. J. Anat. 202, 165–174. 10.1046/j.1469-7580.2003.00144.x 12587931 PMC1571064

